# Structural and functional analysis of TREM2 interactions with amyloid beta reveal molecular mechanisms that drive phagocytosis of oligomeric amyloid beta

**DOI:** 10.1002/alz.092282

**Published:** 2025-01-03

**Authors:** Jessica A Greven, Jennifer M Alexander‐Brett, Thomas J Brett

**Affiliations:** ^1^ Washington University, Saint Louis, MO USA

## Abstract

**Background:**

The development of new innovative treatments to prevent and ameliorate Alzheimer’s disease (AD) requires knowledge of molecular mechanisms that are critical to neuronal health. The receptor TREM2 is part of a signaling complex that modulates inflammatory responses, phagocytosis and cell survival in microglia– resident immune cells in the brain that play a critical role in clearing misfolded aggregates such as amyloid beta (Aβ). In recent years, TREM2 has emerged as a promising drug target for AD. Understanding the molecular mechanisms underlying TREM2 signaling in microglia will facilitate the development of specific, safe and efficacious therapies for AD that target TREM2. With this in mind, we set out to determine the structural mechanism for TREM2 phagocytosis of oligomeric Ab (oAb). We utilized comprehensive structural, biophysical, and functional analysis to achieve this goal.

**Method:**

We used comprehensive biolayer interferometry (BLI) analysis to investigate TREM2 interactions with oAb42 WT and familial variants. We used X‐ray crystallography to determine the structure of TREM2 in complex with an Ab peptide. We used BLI with structure‐guided TREM2 variants to validate the Ab binding site on TREM2. Finally, we used Ab phagocytosis assays in HMC3 microglia cells for functional investigations.

**Result:**

We found that N‐terminal variants in Ab did not bind TREM2. Using this information, we co‐crystallized a short peptide (Ab 1‐8) in complex with TREM2 and determined the structure. We found that it bound at CDR1 hear the hydrophobic site. This was validated by BLI, and mutations to the TREM2 hydrophobic site ablated binding to oAb42. Finally, we found that the interactions between the N‐terminal region of Ab and TREM2 were critical to drive phagocytosis in HMC3 cells.

**Conclusion:**

Our results indicate that TREM2 uses the hydrophobic site (consisting of the CDR1, CDR2, and CDR3 loops) to engage the N‐terminal region of the Ab42 peptide to drive phagocytosis of oAb42. They also suggest that therapeutics that either directly bind or allosterically alter the hydrophobic site on TREM2 should modulate TREM2 signaling as potential AD treatments.

